# Effects of Medium Additives on the Mycelial Growth and Polysaccharide Biosynthesis in Submerged Culture of *Bjerkandera fumosa*

**DOI:** 10.3390/molecules29020422

**Published:** 2024-01-15

**Authors:** Fan Li, Huizhen Fan, Qianwen Sun, Yao Di, Hongmei Xia

**Affiliations:** Engineering Research Center of Glycoconjugates Ministry of Education, School of Life Sciences, Northeast Normal University, Changchun 130024, China; lif885@nenu.edu.cn (F.L.); fanhuizhen@nenu.edu.cn (H.F.); sunqw273@nenu.edu.cn (Q.S.); diy411@nenu.edu.cn (Y.D.)

**Keywords:** medium additives, mycelial biomass, polysaccharide, bioactivity, submerged culture, *Bjerkandera fumosa*

## Abstract

Medium additives have been shown to affect the synthesis of active products in fungi. This study investigated the effects of corn stalk, poplar sawdust, Tween-80, and oleic acid on mycelial biomass and physicochemical properties, as well as the bioactivity of polysaccharides, including exopolysaccharides (EPS) and intracellular polysaccharides (IPS), in the submerged culture of *Bjerkandera fumosa*. Results showed that the addition of corn stalk or poplar sawdust increased the production of EPS but decreased the production of IPS; Tween-80 had less effect on the production of EPS and IPS; and oleic acid stimulated polysaccharide production significantly. Polysaccharide property analysis showed that the addition of corn stalk or poplar sawdust promoted the production of high-molecular-weight components in polysaccharides and changed the monosaccharide composition of polysaccharides, as well as increased the mannose, glucuronic acid, and xylose contents of IPS. Tween-80 and oleic acid also changed the molecular weight distribution of polysaccharides but only slightly affected the composition of monosaccharides. The bioactivity assay indicated that the polysaccharides obtained by adding corn stalk possessed high hydroxyl radical scavenging and antitumor activities. The effect of poplar sawdust was slightly weaker than that of corn stalk. EPS and IPS obtained from a culture with Tween-80 and oleic acid possessed low antioxidant activity. Moreover, their antitumor activity was improved and lost, respectively. The results obtained in this work are useful for improving the understanding of the optimization and regulation of bioactive polysaccharide production in the submerged culture of *B. fumosa*.

## 1. Introduction

Many mushrooms have long been used in traditional therapies due to their rich resources and effective biological activity [1]. Contemporary medical studies have demonstrated that the polysaccharides derived from these macroscopic fungi, mostly higher basidiomycetes and some ascomycetes, are important bioactive constituents and possess numerous bioactivities, such as antioxidant, antitumor, hypoglycemic, and immunomodulatory activities [2,3,4]. In recent years, environmental pollution and human over-picking have made some medicinal fungi increasingly scarce. Attention has been paid to using artificial culturing for the consumption demands, such as solid cultures and submerged fermentation, and the latter has shown great advantages for the production of polysaccharides from medicinal fungi in a compact space and shortened time with reduced chances of contamination [5,6,7]. Furthermore, in culture, it is not always possible to obtain the fruiting bodies of macroscopic fungi found in nature, however it is usually possible to grow these species in fermenters.

In fungi, polysaccharides are synthesized by using intracellular nucleotide sugars at the membrane level, then are either stored in the cytoplasm, used to construct the main structure of the cell wall, or are secreted outside the cell to form a gelatinous matrix. In submerged fermentation, the gelatinous polysaccharides can be further excreted into the growth medium and are referred to as exopolysaccharides (EPSs), while the first two intracellular polysaccharides are referred to as mycelial polysaccharides or intracellular polysaccharides (IPSs) [8]. Current studies have shown that whether microbial polysaccharides have biological activity may be related to their properties and structures, independent of their location, so EPS and IPS are both attractive polysaccharides.

Polysaccharide synthesis is not under direct genetic control; the process is affected by biosynthetic enzymes. So, the polysaccharide production and constituent may be optional depending on the culture medium and growth conditions. Recently, researchers have attached great importance to improving fungal mycelial and polysaccharide yield by a variety of optimization strategies in terms of the fermentation medium or conditions [9,10,11,12]. In particular, the use of some stimulatory agents to improve mycelium growth and product formation has received much attention. Many agents including lignocellulose, plant oils, fatty acids, vitamins, and surfactants have been described to accelerate mycelial growth and polysaccharide prodution in some mushroom species [13,14,15,16,17,18,19,20]. A certain concentration of hemicellulose and lignin was reported to stimulate mycelia growth and polysaccharide biosynthesis in *Lentinus edodes* [13]; olive oil and soybean oil was described to make a higher production of EPS in *Grifola frondosa* [14]; Tween-80 was found to be effective in enhancing the production of bioactive EPS in *Pleurotus tuber-regium* [15]; VB 6 and VB 1 were proved to have a significantly stimulatory effect on the mycelial growth and polysaccharide activity of *Inonotus obliquus* [16]. These stimulatory agents were presumed to increase cell permeability or affect enzymes involved in the synthesis of polysaccharides [17,18,19,20]. However, such studies have generally not addressed the characterization or bioactivity of metabolites. In polysaccharides, bioactivity has been reported to be largely dependent on physicochemical properties, including chemical composition, molecular weight distribution, and monosaccharide composition [21,22]. Studying the intrinsic relationship among fermentation conditions, product characteristics, and product activity is crucial.

*Bjerkandera fumosa* (Pers.) P. Karst is a white-rot fungus which is considered as the main decomposer of dead and fallen trees. It is also a medical fungus that is used in traditional Chinese medicine. The water extract of the fruiting body is considered to be effective in the treatment of uterine carcinoma and the enhancement of immunity. In our previous study, two homogeneous IPS and EPS were purified from mycelium and fermentation broth, respectively, and the results showed both of them had antioxidant and immune modulation activities [23].

In this study, the promoters, namely, lignocellulose, surfactant Tween-80, and oleic acid, mentioned in the previous report were added to the culture medium, and the effects of these additives on the production, properties, and bioactivities of *B. fumosa* polysaccharide were investigated. This work attempted to further understand the effects of exogenous additives on mycelial growth and polysaccharide synthesis by *B. fumosa* in submerged cultures.

## 2. Results

### 2.1. Effects of Medium Additives on Mycelial Biomass and Polysaccharide Production

**Figure 1:**
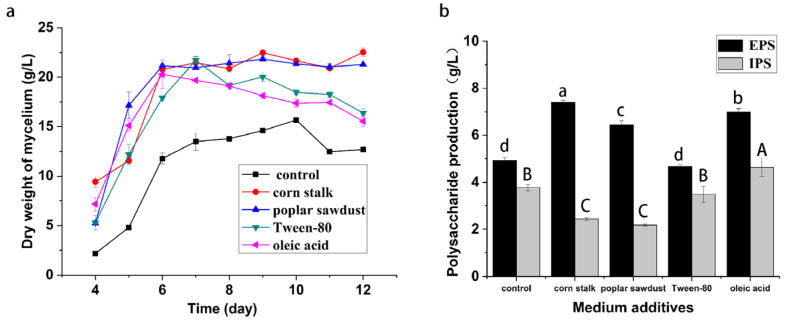
The effect of medium additives on mycelial biomass production (**a**) and polysaccharide yield (**b**) in *B. fumosa*. Differing letters above bars indicate significant (*p* < 0.05) differences.

This study investigated the effects of the medium additives corn stalk, poplar sawdust, Tween-80, and oleic acid on biomass and polysaccharide production of *B. fumosa* in submerged fermentation cultures. As shown in Figure 1, all of the additives could stimulate the growth of *B. fumosa*. The mycelial dry weight obtained after eight days of culture in the medium containing 3% corn stalk or poplar sawdust was approximately 21.5 g/L and that obtained in the medium containing Tween-80 and oleic acid was 19.5 g/L. The results for the production of EPS and IPS by *B. fumosa* showed that the addition of corn stalk or poplar sawdust led to an increased production of EPS but reduced the production of IPS. Adding corn stalk to the medium increased the production of EPS from 4.93 g/L to 7.42 g/L; similarly, the addition of poplar sawdust elevated the production of EPS from 4.93 g/L to 6.45 g/L. However, corn stalk and poplar sawdust significantly reduced the yield of IPS from 3.77 g/L to 2.45 and 2.18 g/L, respectively. The addition of Tween-80 had little effect on the production of EPS and IPS. Consistent with the findings of previous reports on *Grifola frondosa* [14], this result indicated that Tween-80 increased cell growth instead of polysaccharide production. By contrast, oleic acid stimulated the production of polysaccharides significantly. It increased the production of EPS from 4.93 g/L to 6.99 g/L and the production of IPS from 3.77 g/L to 4.63 g/L. The results showed that the additives had effects on biomass and polysaccharide yield in liquid fermentation, but that the effects on both were inconsistent.

### 2.2. Effects of Medium Additives on the Chemical Components of Polysaccharides

The chemical components of polysaccharides, including total carbohydrates, protein, polyphenols, and uronic acid, were measured as described in the Methods section. As shown in Table 1, when corn stalk was added to the medium, the total carbohydrate content decreased, and the uronic acid content increased in EPS and IPS. When poplar sawdust was added, the content of polyphenols decreased and that of uronic acid increased significantly. These results indicated that cellulose and lignin may be involved in the synthesis of polysaccharides, thereby increasing the content of uronic acid. Similar results have also been reported for polysaccharide synthesis in *Lentinula edodes* [13]. However, the specific process of polysaccharide synthesis that involves lignocellulose is unclear.

Table 1 also lists the effects of Tween-80 and oleic acid on the chemical composition of polysaccharides. The results showed that Tween-80 increased the content of total carbohydrates in EPS from 53.12% to 65.13% but had no significant effect on other ingredients. By contrast, oleic acid greatly increased the total carbohydrate content in EPS and IPS and slightly increased the content of uronic acid in polysaccharides.

### 2.3. Effects of Medium Additives on the Molecular Weight Distribution of Polysaccharides

**Figure 2:**
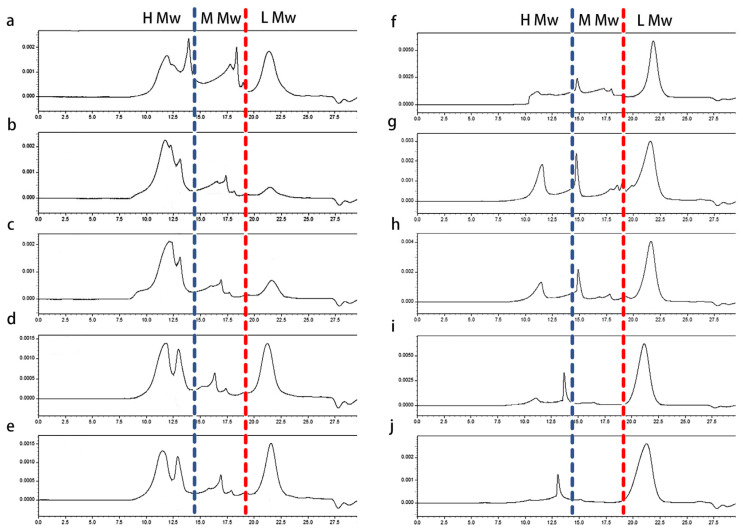
High performance size exclusion chromatography (HPSEC) spectra of IPS (**a**–**e**) and EPS (**f**–**j**) obtained from *B. fumosa* cultured with different mediums. The medium additives were (**a**,**f**) control, (**b**,**g**) corn stalk, (**c**,**h**) poplar sawdust, (**d**,**i**) Tween-80 and (**e**,**j**) oleic acid.

The molecular weight distribution of the polysaccharides obtained from different media were investigated by using high-performance size exclusion chromatography. As shown in Figure 2, IPS and EPS were polydisperse polysaccharides. The average molecular weight of IPS was about 10^3^ kDa and the average molecular weight of EPS was lower, it was found to be 10^2^ kDa. The addition of different additives in the medium changed the proportion of different molecular weight components in polysaccharides.

For the convenience of description, the peaks in the spectrum of polysaccharides were divided into three regions: the high-molecular-weight region (H Mw, molecular weights of more than 500 kDa), low-molecular-weight region (L Mw, molecular weights of less than 10 kDa), and medium-molecular-weight region (M Mw, molecular weights of approximately 10–500 kDa). The ratio of polysaccharides with different molecular weights are summarized in Table 2. Obviously, the addition of corn stalk and poplar sawdust significantly increased the ratio of high-molecular-weight polysaccharides in IPS and EPS, whereas the yield of medium- and low-molecular-weight polysaccharides was reduced. This result indicated that lignocelluloses promoted the synthesis of macromolecular polysaccharides. However, the effect of Tween-80 and oleic acid on polysaccharides was somewhat different from that of corn straw and poplar sawdust. The ratio of high- and low-molecular-weight polysaccharides in IPS increased significantly, but for EPS, it seems that high- and medium-molecular-weight polysaccharides were concentrated in the middle. The above results indicated that the ratio of different molecular weight components in the polysaccharide product varied with the medium supplements, and similar results have also been reported for the effect of some carbon sources on polysaccharides [24,25].

### 2.4. Effects of Medium Additives on the Monosaccharide Composition of Polysaccharides

The monosaccharide composition of polysaccharides was determined via HPLC, and the sample was identified by matching its retention time with the retention times of the standard monosaccharides under the same analytical conditions. As shown in Table 3, EPS and IPS were heteropolysaccharides that were mainly composed of glucose. The glucose percentages of EPS and IPS extracted from *B. fumosa* in basal medium could reach 73.8% and 77.5%, respectively. When corn stalk or poplar sawdust was added to the medium, the proportion of glucose in EPS and IPS decreased, whereas that in most of the other monosaccharide components increased. Specifically, these two additives increased the contents of mannose, galacturonic acid, galactose, and xylose in IPS, and the contents of glucuronic acid and galactose in EPS. However, the effect of poplar sawdust on the monosaccharide composition of EPS was not as significant as that of corn stalk.

The effect of Tween-80 and oleic acid on monosaccharide composition was different from that of the above two additives. They increased the amount of glucose in IPS and reduced the content of rhamnose to undetectable levels. In addition, Tween-80 greatly increased the content of glucose in EPS from 77.5% to 86.7%. This change led to a reduction in the content of other monosaccharide components, except fucose, in heteropolysaccharides. In EPS, oleic acid reduced the glucose content and increased the fucose content significantly from 0.5% to 20.5%. Although the mechanism of this interesting result is not yet clear, it certainly provides us with an effective way to increase the yield of polysaccharides containing fucose.

These results showed that the addition of medium additives not only simply affected the growth of mycelia and the production of polysaccharides, but also changed the composition of polysaccharides significantly. They indicated that these substances and their degradation products participate in complex polysaccharide metabolism, synthesis, and transport processes, resulting in the formation of new heteropolysaccharides.

### 2.5. Effects of Medium Additives on the Antioxidant Activity of Polysaccharides

**Figure 3:**
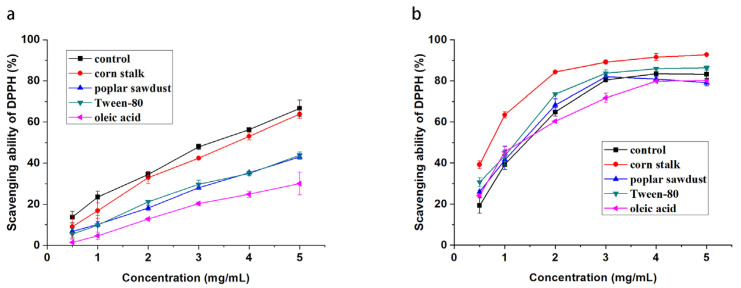
DPPH radical scavenging activity of EPSs (**a**) and IPSs (**b**) from *B. fumosa* cultured with different mediums.

The DPPH radical is a stable free radical with the capability to become a stable diamagnetic molecule by accepting an electron or hydrogen; therefore, it is often used for the assay of free-radical scavenging activities [26,27]. As shown in Figure 3, all EPS and IPS in the samples displayed scavenging activity for DPPH radicals and showed a concentration-dependent increase within the range of 0.5–5 mg/mL. EPS produced by the basal medium culture showed the highest DPPH scavenging activity at each concentration, and its IC50 value could reach approximately 3.2 mg/mL. The activity of EPS produced by adding corn stalk to the medium was similar to that of EPS produced without any additives. However, after the addition of poplar sawdust, Tween-80, and oleic acid to the medium, the DPPH scavenging activity of EPS significantly decreased and reached 43%, 42%, and 30%, respectively, even when the concentration of EPS was 5 mg/mL. Nevertheless, the effect of additives in the medium on the DPPH scavenging activity of IPS was different from that of the DPPH scavenging activity of EPS. IPS had a significantly higher DPPH scavenging activity than EPS in the basal medium, and its IC50 value was 1.5 mg/mL. When corn stalk and Tween-80 were added to the medium, the DPPH scavenging activity of IPS produced from mycelia was promoted. In particular, after the addition of corn stalk, IPS showed significantly increased activity, its IC50 value was reduced by half, and its DPPH scavenging ability reached 90% at the concentration of 3 mg/mL. In contrast, the addition of poplar sawdust had no significant effect. The effect of oleic acid was highly complicated. At low concentrations (0.5–1 mg/mL), the DPPH scavenging activity of mycelial IPS produced in the medium supplemented with oleic acid was higher than that of the control IPS produced in the basal medium. When the concentration was increased to more than 2 mg/mL, the DPPH scavenging activity of the mycelial IPS was lower than that of the control IPS.

**Figure 4:**
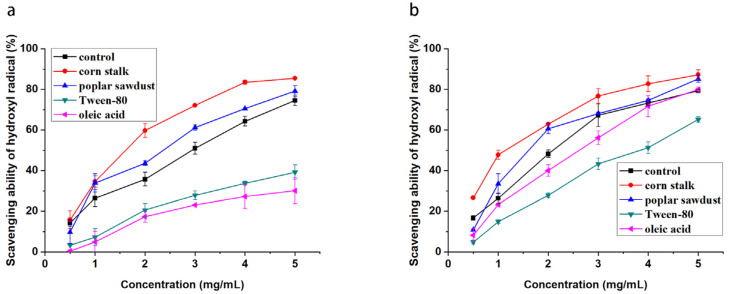
Hydroxyl radical scavenging activity of EPSs (**a**) and IPSs (**b**) from *B. fumosa* cultured with different mediums.

The hydroxyl radical, the most reactive free radical in vivo, attacks all proteins, DNA, PUFA in membranes, and almost any biological molecule it touches [28]. The hydroxyl radical scavenging activity in this study is shown in Figure 4. As shown, all the samples of EPS demonstrated concentration-dependent scavenging activity for hydroxyl radicals. The IC50 value of the hydroxyl radical scavenging activity of EPS derived from the basal culture was 3 mg/mL. The addition of corn stalk and poplar sawdust significantly increased the activity of the produced EPS, and the scavenging activity of 5 mg/mL EPS exceeded 80%. However, the addition of Tween-80 and oleic acid to the medium remarkably reduced the hydroxyl radical scavenging activity of the produced EPS. The IPS samples had high hydroxyl radical scavenging activity. The addition of corn stalk, poplar sawdust, and oleic acid into the culture medium only slightly affected the activity of the produced IPS. By contrast, the addition of Tween-80 into the medium inhibited the hydroxyl radical scavenging activity of IPS and increased the IC50 value from 2 mg/mL to 4 mg/mL.

**Figure 5:**
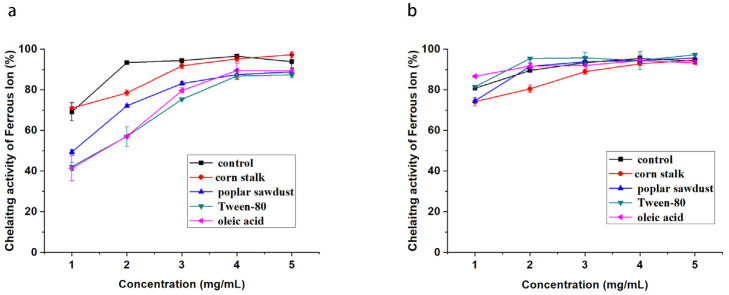
Metal ion chelating activity of EPSs (**a**) and IPSs (**b**) from *B. fumosa* cultured with different mediums.

Metal ion chelating activity has attracted attention because it reduces the concentration of catalytic transition metals in lipid peroxidation. Figure 5 shows the ferrous ion chelating activity of the polysaccharides from *B. fumosa*. As illustrated, EPS derived from the basal culture had very high ferrous ion chelating activity and could exceed 90% at 2 mg/mL. The culture additives, especially poplar sawdust, Tween-80, and oleic acid, could reduce the activity of the produced EPS. The ferrous ion chelating activity of all samples of IPS was high and negligibly affected by the culture additives.

As shown by the results for the three types of antioxidant activities, EPS and IPS from *B. fumosa* had significant antioxidant activity. The activity of IPS was higher than that of EPS. On the whole, the addition of the culture additives had little effect on the IPS and mainly affected the activity of the EPS. The addition of corn stalk and poplar sawdust into the medium reduced the DPPH scavenging activity and ferrous ion chelating activity of the produced EPS but increased hydroxyl radical scavenging activity. These results indicated that the additives may exert a complex effect on the structure of polysaccharides by participating in the anabolism of polysaccharides, which causes different changes in the scavenging abilities of the polysaccharides for different types of oxides. The addition of Tween-80 and oleic acid to the culture medium significantly reduced all the measured antioxidant activities of the produced EPS.

### 2.6. Effects of Medium Additives on the Antitumor Activity of Polysaccharides

**Figure 6:**
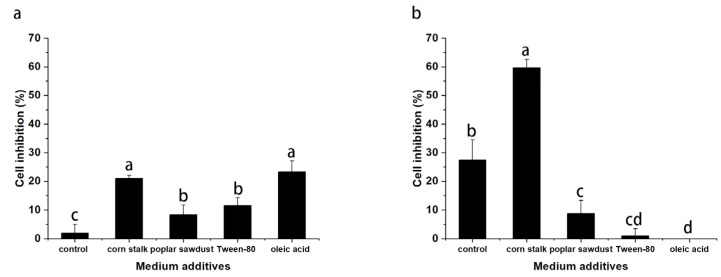
Antitumor activity of EPSs (**a**) and IPSs (**b**) from *B. fumosa* cultured with different mediums. Differing letters above bars indicate significant (*p* < 0.05) differences.

Given that *B. fumosa* is used to treat uterine cancer, in this study, Hela cells were used to test the effects of various additives on the antitumor activity of EPS and IPS. As shown in Figure 6, EPS and IPS inhibited the proliferation of Hela cells, and antitumor activity varied with different additives. All additives promoted the activity of EPS. EPS derived from supplemented media had obvious inhibitory activity at the concentration of 1 mg/mL. The activity of EPS derived from the supplemented media was higher than that of EPS derived from basal medium by 9, 3.6, 5, and 11 times.

However, the effects of different additives on IPS were different. The addition of corn stalk significantly promoted the activity of the produced IPS. At the concentration of 1 mg/mL, IPS derived from the basal medium inhibited the proliferation of Hela cells by 28%. The inhibitory effect of IPS extracted from the mycelia cultured in corn stalk medium could reach 60%. The addition of poplar sawdust, Tween-80, and oleic acid showed a significant inhibitory effect on the activity of mycelial IPS. In particular, the addition of oleic acid caused IPS to lose its inhibitory effect on Hela cells proliferation completely. The specific reason for this effect is still unclear. By combining the analysis results for the properties of polysaccharides and monosaccharide composition, we found that the additives had a more significant effect on the antitumor activity of IPS than on the properties and composition of polysaccharides. This result suggested that the additives may also have a considerable effect on the structure of IPS, thereby remarkably changing its antitumor activity.

## 3. Discussion

Mushroom polysaccharides display a variety of useful functions and have great application value in the food and pharmaceutical industry. The relationship among culture medium, mycelial biomass, and polysaccharide yield has long been an important research topic in the development and improvement of polysaccharide products [12,29]. To accelerate the mycelial growth and polysaccharide production, the modification of media composition and conditions would be vital. In recent years, it has been reported that the incorporation of some specific exogenous stimulants into fermentation medium is favorable to the growth of mycelia in several medicinal mushrooms and increases the production of bioactive metabolites [14,30,31].

In this paper, the effects of two kinds of additives on the mycelia growth and polysaccharide production of *B. fumosa* were studied. Corn stalk and poplar sawdust belong to lignocellulose and given that these substances are more difficult to degrade than soluble monosaccharides and polysaccharides, they may be used as subsequent carbon sources to stimulate the growth of strains. In addition, the degradation products of these substrates may act as signal molecules to participate in the expression regulation of genes involved in sugar transport and polysaccharide biosynthesis, possibly resulting in different polysaccharide yields [13]. Another group includes Tween-80 and oleic acid, which belong to surfactants and fatty acids, respectively, and it has been reported that these substances may promote the uptake of nutrients in the medium by regulating the incorporation of lipids into the cell membrane [32].

Our results demonstrated that these additives effectively promoted the increase in mycelial biomass, but good mycelial growth did not seem to be the determining factor of polysaccharide yield. It was evident that the yield of EPS increased in most additive groups, but that the yield of IPS decreased. Xu et al. evaluated the effect of lignocellulose in wheat straw, rice straw, and sugarcane bagasse on the accumulation of EPS and IPS in *Inonotus obliquus* under submerged fermentation, their results were similar to ours, showing inconsistent effects of additives on mycelial biomass and polysaccharide yield [33]. Yang et al. found that a medium with 2% *Coix Lacryma-Jobi* oil led to an increase in mycelial biomass (3.34-fold) and higher recovery of EPS and IPS (2.2- and 2.23-fold, respectively) in *Ganoderma lucidum* [18]. The addition of coixenolide was reported to increase the biomass, and the EPS and IPS in *Ganoderma lucidum* by 1.39-, 2.58- and 2.24-fold of the control, respectively [34]. Our results of oleic acid group also showed an increase in biomass, EPS, and IPS (1.5-, 1.42- and 1.23-fold, respectively). Based on the above results, it is speculated that the different properties of lignocellulose and fatty acid may determine their different effects on mycelial biomass and polysaccharide yield, indicating that lignocellulose and fatty acid are fundamentally different in the regulation of polysaccharide synthesis. Of course, the factor of strain cannot be excluded, which still needs to be verified by more mushrooms. In terms of mechanism, how the derivation and bioconversion of lignocellulose to the microbe polysaccharides is not fully understood, and it has been suggested that its degradation products may be involved in the regulation of polysaccharide biosynthesis processes, resulting in different polysaccharide yields [13]. Moreover, the stimulatory effect of fatty acids had been proposed to modify membrane composition and increase permeability, or directly affect the synthesis of the enzymes involved in polysaccharide production [17,18,19,20].

The initial carbon source in the medium provided sufficient substrate for polysaccharide synthesis, as specific exogenous additives may affect the composition of polysaccharide by affecting the metabolic flow of the strain or the catalysis of enzymes. Our results of the chemical composition showed that the polysaccharide composition changed with different additives, especially in the group of corn stalk, the total carbohydrates content decreased and the uronic acid increased. The activity experiment also showed that the EPS and IPS of this group had better antioxidant activity and antitumor activity. It has been reported that acidic polysaccharides have a relatively high biological activity, and our results are consistent with this [35,36].

Polysaccharides with different degrees of branching and distinct polymerization will be generated from various mediums. In our study, it can be seen that the molecular weight of EPS and IPS changes significantly after the addition of additives, mainly reflected in the change of polysaccharide in different molecular weight ranges. Current studies on the biological activity of polysaccharides show that low molecular weight polysaccharides may have better antioxidant activity, while high molecular weight polysaccharides are often necessary for polysaccharides to exert antitumor activity [37]. This suggests that we can change the molecular weight of polysaccharides by modifying the medium, so as to change its application direction. The effects of molecular weight changes on the activity of some EPS and IPS in our study were consistent with the reported rules, while some were inconsistent. This may be due to the fact that the polysaccharides produced were not purified, and multi-component crude products will affect the analysis. Meanwhile, we should also be aware that glycans have multiple potential structural elements, in addition to molecular weight, monosaccharide composition, branched chain pattern, and the connection between monosaccharides will affect their activity. This combined effect complicates the study of the structure–activity relationship.

The composition of monosaccharides also changed significantly, compared with the control group, the proportion of glucose in lignocellulose groups decreased while the proportion of other monosaccharides such as mannose increased. Chen et al. studied the submerged fermentation of *Inonotus obliquus* and found that the proportion of mannose in the EPS obtained from the lignocellulose medium was much higher than that from the basal medium, and the proportion of glucose was much lower. The authors speculated that this difference in composition may be one of the reasons for the stronger antioxidant activity of EPS from the lignocellulose medium [38]. Our results of monosaccharide composition and activity analysis were consistent with this report, suggesting that corn stalk may be a good inducer of active polysaccharides for wood-rot fungi.

Additives affected the activities of EPS and IPS, among which, the antioxidant activity and antitumor activity of EPS and IPS from the corn stalk group increased significantly. Similar studies speculated that this may be due to the decomposition of lignocellulose by strains to stimulate mycelium to produce more polysaccharides with higher antioxidant effects to protect themselves [33]. Considering that the molecular weight and monosaccharide composition of polysaccharides were changed by the addition of additives, we believe that this stimulating effect is achieved by changing the structure of polysaccharides, but the specific mechanism needs further study.

Polysaccharides are secondary gene products, which are different with proteins. Therefore, the products from various biosynthetic enzymes in nature are not under strict and direct genetic control in their synthesis [37]. The mechanisms controlling certain biosynthetic events, such as the distribution of branches, the length, and the composition of polysaccharides are not fully elucidated. Therefore, the study of polysaccharides from different strains is very necessary, and further research on the production and characterization of polysaccharides is needed for the development of more functional polysaccharides.

## 4. Materials and Methods

### 4.1. Chemicals

*B. fumosa* and Hela cells were obtained from the China Forestry Culture Collection Center (Beijing, China) and the National Engineering Laboratory for Druggable Gene and Protein Screening (Northeast Normal University, Changchun, China), respectively. Dextran and monosaccharide standards, 1,1-diphenyl-2-picrylhydrazyl (DPPH), thiazolyl blue tetrazolium blue (MTT), and dimethyl sulfoxide (DMSO) were purchased from Sigma-Aldrich LLC. (St. Louis, MO, USA). All other reagents used were of analytical grade.

### 4.2. Strain Cultivation

*B. fumosa* was activated on PDA dextrose agar containing glucose (20 g) and agar (15 g) in 1 L of potato extract at 28 °C for 6 days. Three square pieces of mycelia (0.5 cm^2^) were inoculated into a 500 mL flask containing 200 mL of seed medium (20 g of glucose, 3 g of peptone, 0.1 g of CaCl_2_, 1 g of KH_2_PO_4_, 1.5 g of MgSO_4_, and 2 g of yeast extract in 1 L) and cultured in a rotary shaker at 28 °C and 150 rpm for 6 days. Then, 20 mL of the seed culture was inoculated into a 500 mL flask containing 200 mL of fermentation medium (53 g of corn flour, 3 g of peptone, 0.4 g of K_2_HPO_4_, 1 g of KH_2_PO_4_, 0.5 g of MgSO_4_, 0.5 g of FeSO_4_, 0.02 g of CuSO_4_, 0.01 g of ZnSO_4_, 0.01 g of CoCl_2_, and 0.08 g of MnSO_4_ in 1 L) and cultured in a rotary shaker at 28 °C and 150 rpm for 8 days. Cellulose and lignin media were prepared by adding corn stalk (30 g) and poplar sawdust (30 g) to 1 L of corn flour fermentation medium, respectively. Promoter fermentation media were prepared by adding 400 µL/L of Tween-80 or oleic acid to the corn flour fermentation medium.

### 4.3. Biomass Determination

After cultivation, the mycelia of *B. fumosa* were filtered through gauze and washed with distilled water twice. The biomass was obtained by drying the mycelia at 60 °C to a constant weight.

### 4.4. Preparation of Mycelial and Extracellular Polysaccharides

The mycelia and supernatant were separated from the culture via gauze filtration. After being washed twice, the mycelia were extracted in boiling water for 2 h. This extraction process was repeated three times. All water extracts were combined, precipitated by adding four volumes of 95% ethanol, and then incubated at 4 °C overnight. The precipitate was collected through centrifugation at 10,000 rpm for 20 min and lyophilized as IPS.

The filtrate of the fermentation broth was concentrated by rotary evaporation, then repeatedly frozen and thawed, and centrifuged to remove impurities. The obtained supernatant was also precipitated by adding four volumes of 95% ethanol in accordance with the method described above. The precipitated and lyophilized polysaccharides were collected as EPS.

### 4.5. Determination of Carbohydrate, Protein, and Uronic Acid Contents

The sample was dissolved in water to form a solution of appropriate concentration, and the water-soluble part was used to detect the properties. The total carbohydrate content of polysaccharides was evaluated via the phenol–sulfuric acid spectrophotometric assay with D-glucose as a standard [39]. The protein content of polysaccharides was measured through the Bradford method with BSA as a standard [40]. Uronic acid content was evaluated by using the m-hydroxydiphenyl colorimetric method with D-glucuronic acid as a standard [41].

### 4.6. Analysis of Monosaccharide Composition

A total of 2 mg of the sample was hydrolyzed with 2 mol/L trifluoroacetic acid at 120 °C for 2 h and then derivatized with 1-phenyl-3-methyl-5-pyrazolone. The derivatized sample was applied to an HPLC system (Shimadzu) coupled with a DIKMA Inertsil ODS-3 column (4.6 mm × 150 mm) and SPD-10AVD UV-vis detector. The column was eluted with a mobile phase consisting of acetonitrile and 0.1 mol/L phosphate buffer (pH 7.0, 18.8:81.2, *v*/*v*) at 1.0 mL/min and 35 °C.

### 4.7. Determination of Molecular Weight

The molecular weight distributions of water-soluble IPS and EPS were determined by using a high-performance gel permeation chromatography apparatus (Shimadzu, Kyoto, Japan) equipped with a TSK-G3000 PWXL column (7.8 mm × 300 mm, column temperature 30 °C) and a refractive index detector (Shimadzu, RID-10A). The concentration of the sample solution was 2 mg/mL, and the mobile phase was 0.2 mol/L NaCl with a flow rate of 0.6 mL/min. The standard curve was established by using T-series Dextran (T-10, T-40, T-70, T-500, and T-2000), and the molecular weight of each composition was calculated through comparison with the retention time of the standards [42].

### 4.8. Assay on Antioxidant Activity In Vitro

#### 4.8.1. Assay on DPPH Scavenging

DPPH radical scavenging activity was determined by using the method described by Sharma and Bhat with slight modifications [43]. Briefly, polysaccharide samples were dissolved in distilled water at different concentrations (0.5, 1, 2, 3, 4, and 5 mg/mL). Then, 2 mL of polysaccharide solution was added into 2 mL of DPPH (0.1 M in methanol), and the mixture was shaken and incubated at room temperature in the dark for 30 min before measurement by a spectrophotometer at 517 nm. DPPH scavenging activity was calculated by using the following equation:

Scavenging rate (%) = [OD_0_ − (OD_1_ − OD_2_)]/OD_0_ × 100%, (1)

where OD_0_ is the absorbance of the mixture of DPPH and distilled water, OD_1_ is the absorbance of the sample reaction mixture, and OD_2_ is the absorbance of the mixture of the sample solution and methanol.

#### 4.8.2. Assay on Hydroxyl Radical Scavenging

Hydroxyl radical scavenging activity was determined by using the method reported in the literature [44]. Polysaccharide samples were dissolved in distilled water at different concentrations (0.5, 1, 2, 3, 4, and 5 mg/mL). Then, 1 mL of polysaccharide sample was mixed with 1 mL of 9 mM salicylic acid-ethanol, 1 mL of 9 mM FeSO_4_ solution, and 1 mL of 8.8 mM H_2_O_2_ and incubated at 37 °C for 1 h. After centrifugation at 10,000× *g* for 5 min, the supernatant of the mixture was measured using a spectrophotometer at 510 nm. Hydroxyl radical scavenging activity was calculated by using the following equation:

Scavenging rate (%) = A_0_ − (Ax − Ax_0_) A_0_ × 100%, (2)

where A_0_ is the absorbance of the control with distilled water instead of the sample solution, Ax is the absorbance of the reaction mixture, and Ax_0_ is the absorbance of the control using distilled water instead of H_2_O_2_.

#### 4.8.3. Assay on Fe^2+^ Chelating Activity

Fe^2+^ chelating activity was determined by using the method described by Dong with slight modifications [45]. Polysaccharide samples were dissolved in distilled water at different concentrations (0.5, 1, 2, 3, 4, and 5 mg/mL). A total of 1.0 mL of polysaccharide solution was mixed with 1.0 mL of FeCl_2_ solution (2.0 mmol/L), 0.2 mL of ferrozine solution (5.0 mmol/L), and 2.8 mL of distilled water and incubated at room temperature for 20 min. Then, the absorbance of the mixture was measured at 510 nm. Ascorbic acid was used as the positive control.

The Fe^2+^ chelating activity was calculated by using the following equation:

Chelating activity (%) = [Ab − (Abs − As)]/Ab,(3)

where Ab is the absorbance of the control, which was distilled water instead of the sample; Abs is the absorbance of the reaction mixture; and As is the absorbance of the control using distilled water instead of FeCl_2_.

### 4.9. Assay on Antitumor Activity In Vitro

The antitumor activity of the polysaccharides was evaluated on the basis of the inhibition of the proliferation of Hela cells by using the MTT colorimetric assay method [46]. The Hela cells were seeded into each well of a 96-well plate at a concentration of 1 × 10^4^ cells/mL. After incubation in a 5% CO_2_ incubator at 37 °C for 12 h, the cells were treated with the polysaccharide samples at the concentration of 1 mg/mL. After incubation for 48 h, 20 µL of MTT (5 mg/mL) was added, and the cells were incubated for another 4 h. The MTT-containing medium was then removed, and each well was added with 200 µL of DMSO, shaking for 10 min to dissolve formazan crystals. Absorbance was measured at 570 and 630 nm. The absorbance difference was used as the final absorbance value for calculation.

The inhibition ratio of cell proliferation was calculated by using the following equation:

Inhibition ratio (%) = (ODa − ODb) × 100%/Oda,(4)

where ODa is the absorbance value of the negative control group with untreated cells, and ODb is the absorbance of the sample group.

### 4.10. Statistical Analysis

All experiments were performed in triplicate. Data were expressed as mean ± SD, and differences between means were analyzed using software program IBM SPSS Statistics 25. Letters were used to indicate *p* < 0.05 levels of significance.

## 5. Conclusions

All the tested additives promoted the production of mycelia but had inconsistent effects on the production of polysaccharides. The additives affected the properties of EPS and IPS, especially molecular weight distribution and monosaccharide composition. Among the additives, lignocellulose increased the content of high- and medium-molecular-weight polysaccharides in EPS. Moreover, it increased the content of high-molecular-weight polysaccharides while decreasing that of low- and medium-molecular-weight polysaccharides in IPS. In addition, it changed the monosaccharide composition of polysaccharides, significantly increasing the mannose and xylose content in IPS. The polysaccharides obtained by adding lignocellulose, especially corn stalk, had superior antioxidant and antitumor activities.

The effect of Tween-80 and oleic acid was different from that of lignocellulose. The addition of Tween-80 and oleic acid also changed the molecular weight distribution of polysaccharides but only slightly affected the composition of the monosaccharides. The antioxidant activity of the polysaccharides obtained by adding these substances was inhibited to a certain extent. Although the antitumor activity of IPS was lost, interestingly, that of EPS was greatly improved. This result may be related to the effect of the two substances on the cell membrane.

In summary, we believe that the exogenous substances added into the culture media changed the molecular weight, monosaccharide composition, and structure of polysaccharides by participating in polysaccharide synthesis, thereby affecting polysaccharide activity. Of course, the specific mechanism of this effect requires in-depth research. The present results provide some optimized conditions for the production of biomass and metabolites from *B. fumosa*, as well as experimental information for further studies on metabolism and the structure–function relation of polysaccharides.

## Figures and Tables

**Table 1:** Chemical composition analysis of polysaccharides from B. fumosa *.

	Total Carbohydrates (wt.%)	Protein (wt.%)	Polyphenols (wt.%)	Uronic Acid (wt.%)
IPS-control	40.13 ± 1.03	3.57 ± 0.14	0.18 ± 0.01	8.41 ± 0.76
IPS-corn stalk	33.38 ± 1.44	3.61 ± 0.05	0.18 ± 0.02	9.58 ± 1.51
IPS-poplar sawdust	39.91 ± 2.09	3.65 ± 0.14	0.09 ± 0.01	10.64 ± 1.20
IPS-Tween-80	37.79 ± 0.23	3.68 ± 0.15	0.09 ± 0.01	8.52 ± 0.60
IPS-oleic acid	46.10 ± 1.03	3.62 ± 0.02	0.14 ± 0.02	8.94 ± 0.60
EPS-control	53.12 ± 0.15	3.61 ± 0.14	0.05 ± 0.01	8.62 ± 0.45
EPS-corn stalk	47.79 ± 0.68	3.65 ± 0.01	0.02 ± 0.00	11.49 ± 0.00
EPS-poplar sawdust	53.19 ± 0.34	3.61 ± 0.15	0	11.07 ± 0.30
EPS-Tween-80	65.13 ± 0.42	3.63 ± 0.11	0	8.41 ± 0.76
EPS-oleic acid	71.34 ± 3.50	3.56 ± 0.08	0	9.47 ± 0.45

* Data are expressed as mean ± SD.

**Table 2:** Molecular weight distribution of polysaccharides from *B. fumosa* cultured with different mediums.

	High Mw (%)	Medium Mw (%)	Low Mw (%)
IPS-control	40	28	32
IPS-corn stalk	72	13	15
IPS-poplar sawdust	74	10	16
IPS-Tween-80	60	15	25
IPS-oleic acid	60	15	25
EPS-control	10	10	80
EPS-corn stalk	20	20	60
EPS-poplar sawdust	20	20	60
EPS-Tween-80	20	0	80
EPS-oleic acid	20	0	80

**Table 3:** Monosaccharide composition of the polysaccharides from *B. fumosa* with different mediums.

	Monosaccharide Composition (%) *								
	Man	Rha	GlcA	GalA	Glc	Gal	Xyl	Ara	Fuc
IPS-control	6.0	4.1	1.7		73.8	6.1	2.3	3.7	2.0
IPS-corn stalk	14.1	1.0	2.9	2.1	56.4	9.6	6.7	4.3	2.8
IPS-poplar sawdust	13.5	1.6	3.3	2.6	58.4	8.3	6.6	2.2	3.4
IPS-Tween-80	7.3		1.8		80.3	5.4	1.8	2.3	1.1
IPS-oleic acid	7.2		1.8		76.7	6.0	2.6	2.5	2.5
EPS-control	5.2	1.0		0.5	77.5	4.7	3.9	6.4	0.5
EPS-corn stalk	7.2	1.4	2.9	0.9	62.8	9.1	3.5	5.2	7.2
EPS-poplar sawdust	4.8	1.9	1.0	1.5	71.3	6.4	5.6	5.9	1.5
EPS-Tween-80	3.9				86.7	4.3	1.3	1.7	1.3
EPS-oleic acid	7.1	0.5	0.9		61.1	3.8	2.2	3.8	20.5

* Man: Mannose, Rha: Rhamnose, GlcA: Glucuronic acid, GalA: Galacturonic acid, Glc: Glucose, Gal: Galactose, Xyl: Xylose, Ara: Arabinose, Fuc: Fucose.

## Data Availability

All data generated or analyzed during this study are included in this article.
